# Explainable Machine Learning for Assessing Digital Health Literacy in Older Adults: Validation and Development of a Two-Stage Model Integrating Performance-Based and Self-Assessed Indicators

**DOI:** 10.2196/86171

**Published:** 2026-03-23

**Authors:** Choonghee Park, Jiyeon Park, Seora Kim, Ye Seul Bae, Jae-Heon Kang, Tae-Min Kim, Ji-Won Chun

**Affiliations:** 1 Department of Medical Informatics College of Medicine The Catholic University of Korea Seoul Republic of Korea; 2 The Catholic Medical Center Institute for Basic Medical Science The Catholic University of Korea Catholic Medical Center Seoul Republic of Korea; 3 Big Data Research Center Kangbuk Samsung Hospital Sungkyunkwan University School of Medicine Seoul Republic of Korea; 4 Department of Family Medicine Kangbuk Samsung Hospital Sungkyunkwan University School of Medicine Seoul Republic of Korea

**Keywords:** digital health literacy, eHealth literacy, digital health care, mHealth, machine learning

## Abstract

**Background:**

Digital health literacy (DHL) is the ability to locate, understand, evaluate, and apply health information in digital environments. It is essential for older adults to effectively engage with contemporary health care. However, existing DHL assessments primarily rely on self-reported measures, which are susceptible to subjective bias and often fail to capture actual performance. There is a need for a comprehensive, data-driven approach that integrates objective performance indicators with self-assessments to accurately predict and explain DHL levels in older adults.

**Objective:**

This study develops and validates a machine learning approach to predict DHL levels in older adults by integrating performance-based and self-assessed evaluations.

**Methods:**

We applied a 2-stage methodological framework using 2 independent datasets. In the first stage, to identify performance-based determinants, we assessed actual digital and information comprehension in a separate pilot cohort of 30 older adults (aged 60-74 years). In parallel, to measure self-reported DHL, we conducted an online survey with a distinct group of 1000 older adults (aged 55-74 years) using the Digital Health Literacy Scale and the Korean version of the eHealth Literacy Scale (KeHEALS). Bayesian linear regression was applied to both datasets to identify significant explanatory variables. In the second phase, we trained and validated a binary classification model to predict KeHEALS levels using the survey dataset (n=1000), leveraging the features identified in the first stage. Five machine learning algorithms were evaluated, and the best-performing model was interpreted using Shapley Additive Explanations (SHAP) analysis.

**Results:**

In the pilot performance-based assessment, using a greater number of electronic devices and having higher educational attainment were positively associated with comprehension, whereas alcohol intake showed a negative association. In the self-assessed survey data, key correlates included interest in health-related apps, self-care confidence, age, smoking, alcohol intake, number of devices used, and exercise frequency. Among the machine learning models, categorical boosting demonstrated the most balanced performance (accuracy 0.785, precision 0.769, F1-score 0.765, area under the receiver operating characteristic curve 0.835), outperforming the dummy classifier (accuracy 0.540). SHAP analysis indicated that self-care confidence, health information search, interest in health-related apps, number of electronic devices used, and exercise frequency were the strongest positive contributors to high-DHL predictions, whereas older age and lifestyle factors (alcohol intake, smoking) contributed negatively.

**Conclusions:**

By explicitly integrating performance-based and self-assessed indicators within an explainable machine learning framework, this study demonstrates that DHL in older adults is influenced by both digital engagement and health management factors. These findings suggest that the proposed framework can serve as a structured approach for evaluating DHL in older adults and inform the design of personalized digital health interventions in clinical and community settings.

## Introduction

### Background

The rapid advancement of digital technologies and smart devices has transformed everyday life, particularly in the health care domain. Digital health technologies such as electronic health records, telemedicine, and mobile health (mHealth) apps are increasingly recognized as essential tools for enhancing access to health information, supporting self-management, and improving the efficiency and quality of health care delivery [1]. These technologies empower individuals to better manage their health and facilitate personalized, patient-centered care [2]. Furthermore, community-driven digital health systems have demonstrated their potential to establish sustainable and equitable models of care [3].

However, to fully benefit from digital health technologies, individuals must have a sufficient level of digital health literacy (DHL). DHL refers to the ability to locate, understand, evaluate, and apply health-related information in digital environments to make informed decisions and manage health [4]. Higher DHL levels have been associated with improved health outcomes, including better chronic disease management, engagement in preventive behaviors, increased use of health care services, enhanced quality of life, and reduced risk of depression [5-8]. Furthermore, DHL plays a critical role in narrowing the digital divide and promoting equitable access to health resources across diverse populations.

According to the 2024 Digital Information Divide Survey in Korea, the digital informatization level among older adults corresponded to 71.4% of that observed in the total population. While digital access has largely equalized, reaching 95.3% of the general population’s level, their digital competencies and utilization indices were relatively lower (55.9% and 75.0%, respectively) [9]. These scores are more likely the result of cohort effects and differences in the availability of educational opportunities, health status, and social and technical support, rather than problems caused by aging [10,11]. Consequently, interventions aimed at enhancing DHL in older populations should be evidence-based and sensitive to the diversity of digital experiences in later life, focusing on modifiable barriers related to access, education, and available support rather than chronological age alone.

The majority of existing studies have relied on self-assessment surveys to measure DHL [12-17]. However, such instruments often fail to capture psychosocial dimensions, such as social support, and may not accurately reflect individuals’ actual digital competencies [18]. This underscores the need for a more comprehensive and empirically grounded approach to DHL assessment. Machine learning offers a robust framework for integrating diverse variables, including sociodemographic characteristics, health behaviors, and psychosocial factors, into multidimensional predictive models capable of capturing complex nonlinear interactions [19].

In this study, we employed a Bayesian estimation method to identify key factors associated with DHL-related outcomes, specifically leveraging a wide range of input features, including psychosocial attributes. Subsequently, we developed machine learning models to predict DHL levels and applied Shapley Additive Explanations (SHAP), an explainable artificial intelligence technique, to enhance the interpretability and transparency of the model’s outputs [20]. This 2-stage approach aims to provide a more accurate and comprehensive understanding of DHL in older adults and offers practical insights into the design of personalized digital health education and intervention strategies.

### Assessment Tool for Measuring Digital Health Literacy: eHealth Literacy Scale

Norman and Skinner’s [21] foundational work conceptualized eHealth literacy as a composite of 6 core literacies: traditional literacy, information literacy, media literacy, health literacy, scientific literacy, and computer literacy. This framework is grounded in the “Lily Model,” which categorizes skills into analytical and context-specific domains. As digital health technologies have advanced, the original construct of eHealth literacy has broadened and is now more commonly used within the wider notion of DHL.

At the same time, several conceptual and methodological limitations of the eHealth Literacy Scale (eHEALS) have been identified and should be considered when the instrument is used. As a self-report measure, eHEALS assesses subjective confidence rather than objectively measured performance, so scores may diverge from actual digital and health-related skills [22,23]. Factor-analytic studies have also reported inconsistent dimensional structures across populations and languages, indicating that the underlying constructs may vary by cultural and demographic context and that direct score comparisons or universal cutoffs should be interpreted with caution [24]. Nevertheless, eHEALS has been translated and validated in numerous countries, demonstrates consistently high internal consistency, and remains a pragmatic core instrument for assessing perceived eHealth literacy in large-scale surveys and clinical research [24-33].

Using eHEALS, Berkowsky [17] assessed the DHL levels of adults aged 65 and older in California and examined the impact of sociodemographic characteristics and digital usage patterns. Regression analyses indicated that higher educational attainment, more frequent internet use, and engagement in a wider range of online activities (eg, email, online shopping, social media) were associated with higher DHL levels, whereas basic demographic variables such as age, gender, and income were not significant predictors. These findings suggest that actual digital engagement may be more critical than demographic background in shaping perceived DHL.

Similarly, Kyaw et al [34] investigated DHL among 434 adults aged 65 and older in urban settings, focusing on the influence of social and digital factors. Their findings revealed that older age was negatively associated with DHL, whereas higher income, extended internet use, and active participation in social media showed positive associations. Notably, older adults who used social media exhibited approximately 4-fold higher DHL levels than nonusers, and those using the internet for more than 3 hours per day demonstrated up to a 35-fold increase in DHL levels. These findings suggest that improving DHL among older adults requires not only the provision of digital technologies but also strategies to promote real-world digital engagement and enhance social participation in digital environments.

### Assessment Tool for Measuring Digital Health Literacy: Digital Health Literacy Scale

Early definitions of eHealth literacy focused primarily on individuals’ abilities to search for and understand health information on the internet. However, today’s digital environment increasingly demands interactive engagement through platforms such as social media, mHealth apps, and telemedicine. Consequently, the concept has evolved into DHL, which encompasses not only information seeking but also the skills to evaluate, apply, and interact with digital health content across diverse platforms [35].

In line with this expanded conceptualization, the Digital Health Literacy Scale (DHLS) was developed as a multidimensional tool to comprehensively measure DHL. The DHLS evaluates competencies across 4 domains: use of digital devices, understanding of health information, use and decision of health information, and use intention [36]. The use of digital devices domain assesses individuals’ proficiency in operating digital tools and accessing health-related services. The understanding of health information domain measures the ability to comprehend online content, such as medication instructions and test results. The use and decision of health information domain evaluates both the application of health information in decision-making and the critical judgment of its credibility. The use intention domain captures users’ motivation and willingness to engage with digital health platforms and services. It was specifically designed to reflect the behavioral, cognitive, and motivational characteristics commonly observed in older adults, particularly those vulnerable to the digital divide. By independently assessing both digital and health literacy and integrating them into a unified DHL construct, the DHLS facilitates a comprehensive and multidimensional assessment of digital health literacy. This approach helps identify differences in digital health literacy across various skill domains, from basic digital operations to advanced critical thinking, providing actionable insights into the specific competencies that require support among older adults.

### Machine Learning Approach for Assessing Digital Health Literacy

Given what we discussed so far, eHEALS and DHLS are widely used self-report measures for evaluating digital health literacy. Most studies employing these tools use univariate analyses to examine the effects of individual variables independently [37-39]. However, because self-assessment instruments are inherently grounded in subjective perceptions, they have substantial limitations in capturing actual performance and real-world information behaviors. This concern aligns with extensive evidence from cognitive and behavioral research demonstrating that self-assessments are often systematically biased and display only modest associations with objective indicators of competence [40]. Moreover, they fail to account for interactions among variables or evaluate the relative importance of each factor [41,42]. These limitations emphasize the need to develop data-driven approaches that use diverse individual-level characteristics to accurately predict DHL.

To address these limitations, this study adopted a comprehensive 2-stage machine learning framework that integrates performance-based and self-assessed indicators of DHL. Machine learning is a statistical approach that predicts and classifies individual conditions by learning complex, nonlinear patterns from multidimensional data [43]. Research suggests that machine learning algorithms, including Extreme Gradient Boosting (XGBoost), random forest, and logistic regression, can identify key sociodemographic factors influencing general health literacy [44]. Existing studies have demonstrated the effectiveness of machine learning in uncovering intricate variable interactions and enhancing predictive accuracy [45-47], reflecting a broader shift toward objective data-driven assessment in health literacy research.

**Figure 1:**
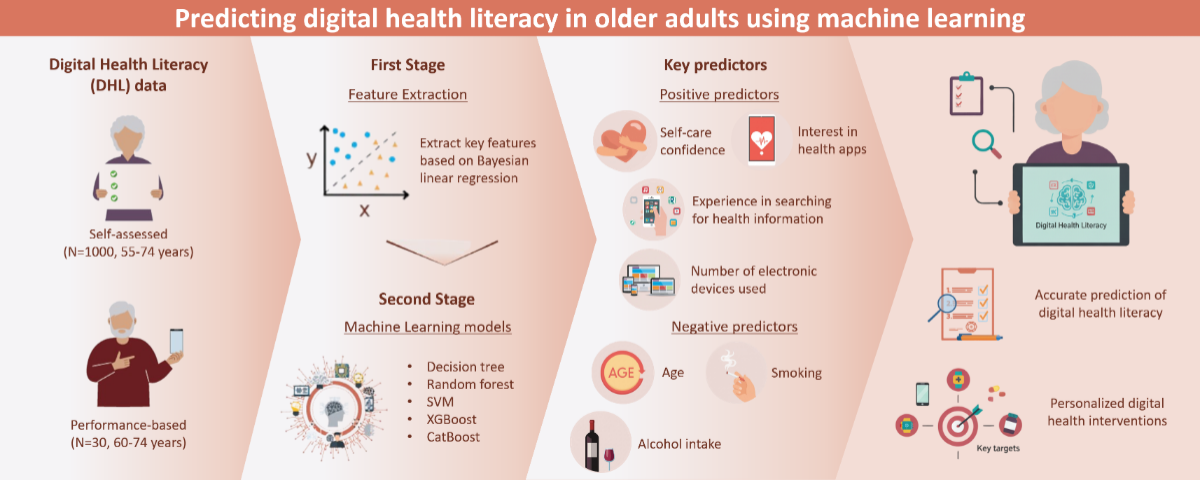
Graphical abstract.

This study integrated performance-based and self-reported assessments to identify key features associated with DHL. Based on these significant features, a prediction model was developed using a machine learning algorithm. Furthermore, we used SHAP to interpret the contribution of each feature, thereby proposing an explainable artificial intelligence approach for medical apps. This approach aims to effectively predict DHL in older adults and provide a foundation for developing personalized intervention strategies (Figure 1).

## Methods

### Overview

**Figure 2:**
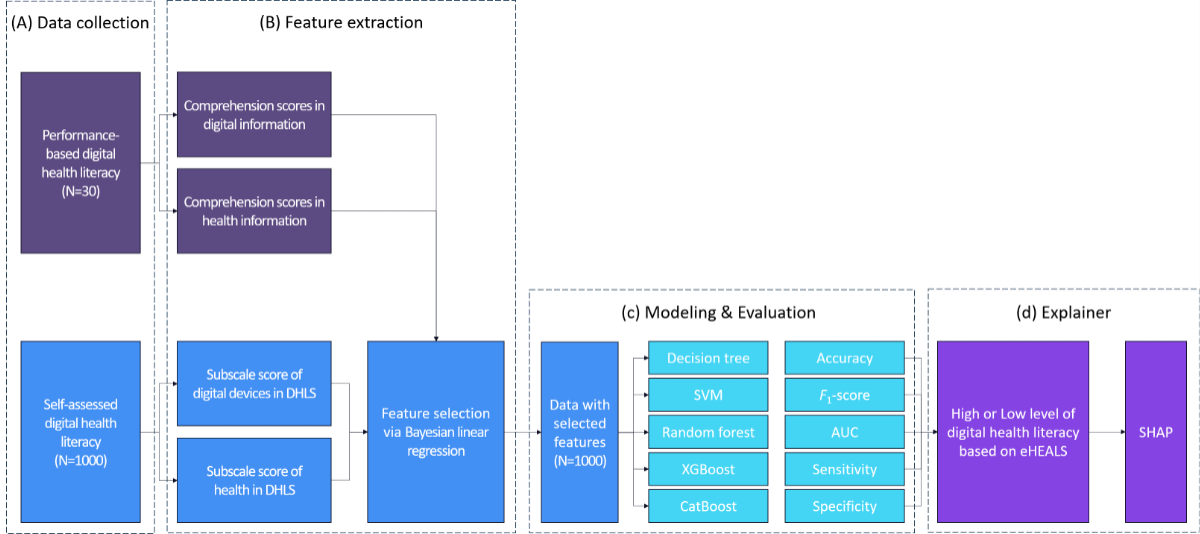
Overview of the framework used to predict digital health literacy level. AUC: area under the receiver operating characteristic; CatBoost: categorical boosting; DHLS: Digital Health Literacy Scale; eHEALS: eHealth Literacy Scale; SHAP: Shapley Additive Explanations; SVM: support vector machine; XGBoost: Extreme Gradient Boosting.

In this study, we designed a 2-stage machine learning framework integrating self-assessed (n=1000) and performance-based (n=30) evaluations (Figure 2). In the initial phase, using a performance-based task, digital literacy and health care information scores were obtained (Figure 2A). Subsequently, Bayesian linear regression analysis was performed to identify explanatory variables significantly associated with DHL (Figure 2B). The performance-based evaluation was employed to enhance the explanatory power of the variables and was excluded from the training phase of the DHL predictive model. In the second phase, using a dataset of 1000 self-assessments and the characteristics identified in the initial phase, we constructed prediction models to classify DHL levels (high vs low; Figure 2C). We conducted comparative evaluations of multiple machine learning models, including decision tree, support vector machine (SVM), random forest, XGBoost, and categorical boosting (CatBoost). In addition, SHAP analysis was used to quantitatively assess the impact of significant variables on DHL predictions, thereby enhancing the interpretation of the model’s predicted results (Figure 2D).

### Performance-Based Digital Health Literacy

#### Participants

To evaluate performance-based DHL, we recruited a total of 30 participants aged 60-74 (mean age 69.1 years). Participants were enrolled using a nonprobability snowball sampling strategy facilitated through local community networks. To ensure data validity and minimize potential bias, intermediaries who arranged the initial contact were excluded from participation. Trained research staff (CP and SK) conducted a brief telephone screening to confirm eligibility and then scheduled a home visit. During the visits, researchers provided comprehensive information regarding the study’s purpose, procedures, anonymity, data usage, and participants’ rights. Written informed consent was obtained before administration of the behavioral tasks. Participation was voluntary, and all participants were informed that they could withdraw from the study at any time without penalty.

To examine differences in performance-based DHL, participants were divided into 2 groups using the eHEALS cutoff score as the criterion: low DHL (n=15) and high DHL (n=15), based on their Korean version of the eHealth Literacy Scale (KeHEALS) scores, as shown in Table 1 [48]. The 2 groups showed no significant differences in age (mean age: low DHL=68.7 years, high DHL=69.4 years; *P*=.19) or gender (male/female: low DHL=2/13, high DHL=5/10; *P*=.34). In addition, no significant difference was found between the groups in years of education (*P*=.49).

**Table 1:** Comparison of performance-based sociodemographic variables by DHL^a^ level based on the KeHEALS^b^ score.

Characteristics			Groups n (%)				Total n (%)
			Low		High		
**Gender**							
	Male	2 (29)		5 (71)		7 (100)	
	Female	13 (57)		10 (43)		23 (100)	
**Age (years)**							
	60-64	1 (33)		2 (67)		3 (100)	
	65-69	9 (64)		5 (36)		14 (100)	
	70-74	5 (38)		8 (62)		13 (100)	
**Years of education**							
	Elementary school	3 (60)		2 (40)		5 (100)	
	Middle school	3 (75)		1 (25)		4 (100)	
	High school	8 (57)		6 (43)		14 (100)	
	Community college	0 (0.0)		1 (100)		1 (100)	
	University or higher	1 (17)		5 (83)		6 (100)	

^a^DHL: digital health literacy.

^b^KeHEALS: Korean version of the eHealth Literacy Scale.

#### Measurements

Participants engaged in content reading focused on digital literacy and health care information, followed by an identification task designed to assess comprehension. Each reading selection comprised 2 or 3 paragraphs and was displayed on a Samsung Galaxy A15 smartphone equipped with a 163.9-mm display (1080 × 2340 pixels). The smartphone was used as the experimental tool to deliver all visual materials for task performance in a controlled viewing environment. During the identification task, participants were asked to indicate whether 10 digital literacy sentences and 10 health care information sentences derived from the readings were true or false. The average time required to complete the assessment did not exceed 30 minutes. To identify key explanatory variables, scores from this identification task were subsequently used in the initial stage of the DHL framework. Before the assessment, demographic information (gender, age, and years of education), psychosocial factors (living arrangements), digital-related factors (interest in health-related apps, health information search, and number of digital devices used), and health-related factors (alcohol intake, smoking, and exercise frequency) were collected from each participant. Interest in health-related apps was measured using a 5-point Likert scale (1=strongly disagree to 5=strongly agree), capturing the degree of interest in using health-related apps in digital environments. Health information search was assessed using a binary (yes/no) item asking whether participants had personally searched for health-related information.

To evaluate participants’ overall level of digital health literacy, the KeHEALS was administered. KeHEALS is an 8-item self-report instrument designed to measure individuals’ perceived ability to locate, evaluate, and apply electronic health information, demonstrating high internal consistency (Cronbach α=.89) and good test-retest reliability over a 2-week interval (intraclass correlation 0.80). Each item was rated on a 5-point Likert scale ranging from 1 (strongly disagree) to 5 (strongly agree), yielding a total score between 8 and 40, with higher scores indicating greater perceived digital health literacy. Participants were classified into 2 groups using a sample-specific threshold based on the median total eHEALS score of 29 in the entire sample: those with a total score greater than 29 were categorized as having a high level of digital health literacy, whereas those with a score of 29 or below were categorized as having a low level. This classification facilitated subsequent analyses by enabling group comparisons according to participants’ perceived competencies in engaging with digital health information.

### Self-Assessed Digital Health Literacy

#### Participants

To evaluate self-assessed DHL for the predictive modeling phase, survey data were collected from 1000 adults aged 55-74 (mean age 63.6 years). All participants were recruited through an online survey platform operated by a survey agency. Recruitment was facilitated via email and website postings. To ensure a representative sample of the general population, stratified quota sampling was employed based on gender and age groups. Each respondent was assigned a unique access link to ensure single participation and eliminate the possibility of repeated submissions. Given the prospective and anonymous nature of this large-scale survey, the requirement for written informed consent was waived by the Institutional Review Board. Participants were first presented with information on the initial survey screen that provided comprehensive details about the study objectives and procedures. Proceeding to the questionnaire was considered to indicate informed willingness to participate.

To examine group-level differences in DHL, participants were classified into 2 groups based on KeHEALS scores: low DHL (n=563) and high DHL (n=437; Table 2). The high-DHL group had a greater proportion of participants in the younger age range of 55-59 (148/437, 33.9%), whereas the low-DHL group included more participants in the 65-69 (242/563, 43.0%) and 70-74 (80/563, 14.2%) age ranges. Age distribution also differed between the groups (mean age: low DHL=64.2 years, high DHL=62.8 years). Differences were likewise observed in gender distribution (male/female: low DHL=257/306, high DHL=243/194). The proportion of males was higher in the high-DHL group (243/437, 55.6%) than in the low-DHL group (257/563, 45.6%), whereas females constituted the majority in the low-DHL group (306/563, 54.4%). Regarding years of education, a marked difference was found between the 2 groups. In the high-DHL group, more than half of the participants (235/437, 53.8%) had obtained a university degree or higher, whereas the low-DHL group had a greater proportion of high school graduates (271/563, 48.1%) and individuals with less than a middle school education.

**Table 2:** Comparison of self-assessed sociodemographic variables by DHL^a^ level based on the KeHEALS^b^ score.

Characteristics		Groups, n (%)		Total, n (%)
		Low	High	
**Gender**				
	Male	257 (51.4)	243 (48.6)	500 (100)
	Female	306 (61.2)	194 (38.8)	500 (100)
**Age (years)**				
	55-59	134 (47.5)	148 (52.5)	282 (100)
	60-64	107 (57.5)	79 (42.5)	186 (100)
	65-69	242 (57.9)	176 (42.1)	418 (100)
	70-74	80 (70.2)	34 (29.8)	114 (100)
**Education**				
	Elementary school	5 (83.3)	1 (16.7)	6 (100)
	Middle school	45 (93.8)	3 (6.3)	48 (100)
	High school	271 (66.4)	137 (33.6)	408 (100)
	Community college	62 (50.4)	61 (49.6)	123 (100)
	University or higher	180 (43.4)	235 (56.6)	415 (100)

^a^DHL: digital health literacy.

^b^KeHEALS: Korean version of the eHealth Literacy Scale.

#### Measurements

DHL levels were assessed using the DHLS [36] and KeHEALS [48]. The DHLS consists of 4 subscales: use of digital devices (10 items), understanding of health information (5 items), use and decision of health information (5 items), and use intention (5 items). Participants completed the DHLS questionnaire using a 5-point Likert-type response scale (1=strongly disagree, 5=strongly agree). To determine key explanatory variables related to digital literacy and health care information in older adults, we focused on 3 subscales: use of digital devices, understanding of health information, and use and decision of health information. Based on a sample-specific threshold corresponding to the median total eHEALS score of 29, eHEALS was dichotomized into 2 groups and used as a predictive variable in the machine learning model. Furthermore, we collected demographic information (gender, age, and years of education), psychosocial factors (living arrangements and social support), digital-related factors (interest in health-related apps, health information search, and number of digital devices used), and health-related factors (alcohol intake, smoking, exercise frequency, and self-care confidence). Self-care confidence was assessed as an indicator of health empowerment in older adults using a single item derived from the Korean Health Empowerment Scale, which measures confidence in making health management decisions [49]. This item was rated on a 5-point Likert scale (1=strongly disagree to 5=strongly agree).

### Statistical Analysis

#### Feature Extraction Based on Bayesian

To identify the most influential factors associated with DHL, we applied Bayesian linear regression to both performance- and survey-based datasets. This approach improves modeling efficiency while enhancing interpretability by incorporating prior-based regularization and probabilistic estimation of regression coefficients, thereby allowing direct assessment of variable importance and uncertainty [50]. Each model was specified separately for each observation unit as follows:



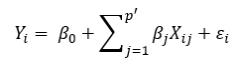



where *Y_i_* is the standardized score of the *i*th participant; *X_ij_* is the value of the *j*th feature for the *i*th participant; *β*_0_ is the intercept; *β_j_* is the regression coefficient for each feature; *ε_i_* is the error term; and *p*′ is the total number of features considered.

The likelihood for the observed data was specified as follows:



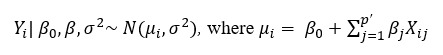



We assigned weakly informative priors to the model parameters to ensure that posterior inferences were primarily driven by the observed data. Specifically, the intercept term *β*_0_ was assigned a prior distribution *N*(0, 5)^2^, and each regression coefficient *β_j_* received an independent prior *N*(0, 1^2^) for *j*=1, ..., *p*′, reflecting an implicit L2 regularization effect. The SD of the error term *σ* was assigned a HalfCauchy(2) prior.

Posterior distributions were approximated using Markov chain Monte Carlo sampling. For each model, 4 parallel chains were run, each with 2000 tuning steps followed by 2000 sampling steps. Convergence was assessed by monitoring divergence diagnostics throughout the sampling process.

Feature selection was conducted using a Bayesian inference framework. The dataset was first split into training and test sets, and all feature selection procedures were performed exclusively on the training set. In the performance-based dataset, we separately identified predictors of comprehension scores for digital information and health information. In the self-assessed dataset, feature selection was performed for each DHLS subscale, namely, use of digital devices and health information understanding and utilization. To prevent data leakage during the Markov chain Monte Carlo sampling procedure used for feature selection, all modeling steps were conducted exclusively on the training dataset. Variables were retained only if the 95% highest density interval (HDI) of their posterior regression coefficients *βⱼ* did not include 0, indicating a statistically significant positive or negative association with the outcome variable. These statistically validated features were then used exclusively in subsequent stages to construct the final predictive model for DHL.

#### Modeling

In this study, final predictive modeling was performed using the self-assessed DHL dataset comprising 1000 participants. The goal of the model was to accurately classify participants into 2 distinct groups: high DHL and low DHL. Before developing the final predictive model, a feature selection process was conducted. This process aimed to identify features strongly associated with both performance-based and self-assessed DHL measures. The resulting feature set was designed to comprehensively represent the various aspects of DHL.

Using this selected feature set, 5 machine learning models—decision tree, random forest, XGBoost, CatBoost, and SVM—were implemented to predict DHL levels [51-55]. During data preprocessing, we applied a structured workflow to handle distinct variable types and used mixed encoding strategies to reflect the measurement properties of each predictor. Nominal categorical variables were encoded using OneHotEncoder. Ordinal variables were generally processed with OrdinalEncoder to preserve their inherent ranking. However, for selected lifestyle variables such as alcohol intake, we applied OneHotEncoder to capture category-specific associations across frequency levels that may not be adequately represented by a single ordered scale. These transformations were integrated into a unified preprocessing pipeline and applied consistently across all data splits, ensuring uniform feature representation and minimizing the risk of data leakage.

For model training and testing, the preprocessed dataset was split into a training set (n=800) and a test set (n=200). The hyperparameters of each model were optimized using the Optuna framework [56], with 10-fold cross-validation applied to the training set to maximize accuracy. Following hyperparameter optimization, to assess generalization performance, each final model was trained on the entire training set and evaluated on the test set.

#### Performance Evaluation

Model performance in predicting DHL levels was quantitatively evaluated using standard metrics for binary classification, including accuracy, precision, sensitivity, specificity, *F*_1_-score, and area under the receiver operating characteristic curve (ROC AUC). Accuracy indicates the overall proportion of correctly classified cases. Precision reflects the proportion of true high-DHL cases among those predicted to have high DHL. The *F*_1_-score, which is the harmonic mean of precision and recall, provides a balanced metric that is particularly useful under class imbalance. The ROC AUC assesses the model’s ability to distinguish between high- and low-DHL cases across varying decision thresholds. Collectively, these metrics offer a comprehensive evaluation of model performance.

Furthermore, to address the “black box” nature of machine learning and ensure clinical transparency, we used the SHAP framework. SHAP is a model-agnostic method based on cooperative game theory that assigns each feature an importance value for a particular prediction. It calculates the Shapley value, which represents the average marginal contribution of a feature across all possible coalitions of features [20]. Unlike traditional feature-importance metrics that provide only a global ranking, SHAP offers both global and local interpretability. It allows us to quantify not only the magnitude but also the direction of each feature’s impact on the model’s output.

In the context of our model for predicting digital health literacy, SHAP provides a granular explanation of how specific demographic, interest in digital health care, health management, and psychosocial features influence the classification of an individual into the high-DHL or low-DHL group. This methodology enables visualization of individual and global feature effects, allowing identification of key determinants of DHL and verification of whether the model’s learned patterns are consistent with established clinical research on digital health.

### Ethical Consideration

This study was reviewed and approved by the Institutional Review Board of the Catholic University of Korea, College of Medicine (survey study approval number MIRB 20230825-007; behavioral task study approval number MIRB 20241014-003), and was conducted in accordance with institutional and national guidelines for research involving human participants. For the web-based survey, an online written information sheet described the study purpose, procedures, minimal-risk nature, anonymity, data use, and participants’ rights. Data were collected only from individuals who voluntarily agreed to participate after reading this information, with explicit notice that they could withdraw from the survey at any time without penalty. For the behavioral task study, participants received a written information sheet and provided written informed consent before participation. In the survey study, no personally identifiable information was collected. In the behavioral task study, personal information was collected solely for compensation purposes, stored separately from research data, and destroyed immediately after compensation. In both studies, no personal identifiers were used for analysis or reporting; all analytic datasets were anonymized, encrypted, and stored on secure institutional servers accessible only to the research team. Participants who completed the web-based survey received 500 points (approximately US $0.35) redeemable within the online platform, and participants in the behavioral task study received KRW 30,000 in cash (approximately US $20) as compensation for their time. No identifiable images of participants were included in the manuscript or its multimedia appendix.

## Results

### Post Hoc Age-Group Comparison of Selected Features

To verify the validity of applying selected features from a small cohort of individuals aged 60 years and older to the 55-59 age group included in the final model during the feature-extraction phase, we divided participants into 2 age groups and performed statistical comparisons of the selected features. The comparison revealed significant demographic and behavioral differences in several variables (Table 3). Modest but statistically significant age-related differences were observed for interest in health-related apps (*P*=.03), health information search (*P*=.02), smoking (*P*=.004), and alcohol intake (*P*=.007). However, no significant differences were observed in the number of electronic devices used (*P*=.41), self-care confidence (*P*=.19), or exercise frequency (*P*=.86).

**Table 3:** Comparison of self-assessed digital and health-related factors by age groups.

Comparison		Age groups (years), n (%)			Chi-square (*df*)			Cramer *V*			*P* value	
		55-59 (n=282)	60-74 (n=718)									
**Interest in health-related apps**						10.388 (4)			0.102			.03
	Strongly disagree	14 (5.0)	76 (10.6)									
	Disagree	46 (16.3)	140 (19.5)									
	Neither agree nor disagree	75 (26.6)	169 (23.5)									
	Agree	86 (30.5)	196 (27.3)									
	Strongly agree	61 (21.6)	137 (19.1)									
**Health info search**						5.617 (1)			0.075			.02
	Yes	224 (79.4)	518 (72.1)									
	No	58 (20.6)	200 (27.9)									
**Number of electronic devices used**						2.902 (3)			0.054			.41
	1	100 (35.5)	278 (38.7)									
	2	111 (39.4)	291 (40.5)									
	3	58 (20.6)	116 (16.2)									
	4	13 (4.6)	33 (4.6)									
**Self-care confidence**						6.095 (4)			0.078			.19
	Strongly disagree	0 (0.0)	5 (0.7)									
	Disagree	23 (8.2)	58 (8.1)									
	Neither agree nor disagree	101 (35.8)	273 (38.0)									
	Agree	151 (53.5)	347 (48.3)									
	Strongly agree	7 (2.5)	35 (4.9)									
**Exercise frequency per week**						1.314 (4)			0.036			.859
	0	38 (13.5)	81 (11.3)									
	1	104 (36.9)	259 (36.1)									
	2	63 (22.3)	165 (23.0)									
	3	49 (17.4)	133 (18.5)									
	4	28 (9.9)	80 (11.1)									
**Smoking**						13.192 (3)			0.115			.004
	Never smoke	158 (56.0)	371 (51.7)									
	Former smoker	66 (23.4)	244 (34.0)									
	Occasional smoker	11 (3.9)	23 (3.2)									
	Heavy smoker	47 (16.7)	80 (11.1)									
**Alcohol intake**						17.904 (6)			0.133			.007
	Never drink	26 (9.2)	110 (15.3)									
	Abstinence in the past year	33 (11.7)	120 (16.7)									
	Less than once a month	52 (18.4)	147 (20.5)									
	About once a month	38 (13.5)	90 (12.5)									
	2-4 times a month	84 (29.8)	152 (21.2)									
	2-3 times a week	36 (12.8)	69 (9.6)									
	4 or more times a week	13 (4.6)	30 (4.2)									

### Feature Selection From Performance-Based Digital Health Literacy

Feature selection using Bayesian linear regression identified 3 features that were statistically associated with digital information comprehension. The number of electronic devices used showed a strong positive association with comprehension scores (β=.59, 95% HDI 0.21-0.93). By contrast, 2 variables related to alcohol intake were negatively associated with comprehension. Specifically, alcohol intake once a month (β=–1.48, 95% HDI –2.43 to 0.49) and alcohol intake 2-4 times a month (β=–1.05, 95% HDI –1.76 to 0.35) were associated with lower performance in digital literacy tasks.

For health care information comprehension, 2 features were statistically significant. Years of education showed a positive association with comprehension performance (β=.48, 95% HDI 0.10-0.88), indicating that higher educational attainment contributes to better understanding of health-related information. Conversely, alcohol intake 2-4 times a month was again associated with reduced performance (β=–1.03, 95% HDI –1.96 to 0.16). Finally, the features selected from the performance-based DHL were the number of electronic devices used, alcohol intake, and years of education. These selected variables were subsequently used in the second-stage predictive modeling of DHL levels.

### Feature Selection From Self-Assessed Digital Health Literacy

The significant predictors associated with 2 DHLS subscales, namely, use of digital devices and health information understanding and utilization, were identified using Bayesian linear regression.

For the digital device utilization subscale, 12 features were statistically significant. Interest in health-related apps was the strongest positive predictor (β=.44, 95% HDI 0.39-0.48). Moreover, several health behavior variables showed positive associations, including abstinence from alcohol intake in the past year (β=.31, 95% HDI 0.09-0.51), alcohol intake about once a month (β=.37, 95% HDI 0.15-0.57), alcohol intake 2-4 times a month (β=.31, 95% HDI 0.11-0.49), alcohol intake 2-3 times a week (β=.26, 95% HDI 0.04-0.52), and being a nonsmoker (β=.19, 95% HDI 0.001-0.37). In addition, self-care confidence (β=.13, 95% HDI 0.03-0.26) was positively associated with digital device use. By contrast, several variables demonstrated significant negative associations with digital device use, including age 70-74 years (β=–.31, 95% HDI –0.50 to 0.11), age 65-69 years (β=–.17, 95% HDI –0.31 to 0.05), number of electronic devices used (β=–.19, 95% HDI –0.26 to 0.13), exercise frequency (β=–.11, 95% HDI –0.17 to 0.05), and years of education (β=–.07, 95% HDI –0.14 to 0.01). Distributions of digital literacy scores by feature values are presented in Multimedia Appendix 1.

For the subscale on health information understanding and utilization, 7 predictors showed statistically significant associations. Interest in health-related apps emerged as the strongest positive predictor (β=.34, 95% HDI 0.27-0.38). Lack of health information search (β=.17, 95% HDI 0.02-0.32), abstinence from alcohol intake in the past year (β=.28, 95% HDI 0.07-0.55), and former smoking status (β=.34, 95% HDI 0.03-0.69) also showed positive associations with health information understanding and utilization. By contrast, negative associations were observed for the number of electronic devices used (β=–.14, 95% HDI –0.21 to 0.07), age 70-74 years (β=–.29, 95% HDI –0.54 to 0.09), and age 65-69 years (β=–.25, 95% HDI –0.41 to 0.11). These variables, including digital information interest and health behavior factors selected from the self-assessed DHL, were subsequently used as input features in the modeling phase.

### Predictive Modeling of Digital Health Literacy

**Figure 3:**
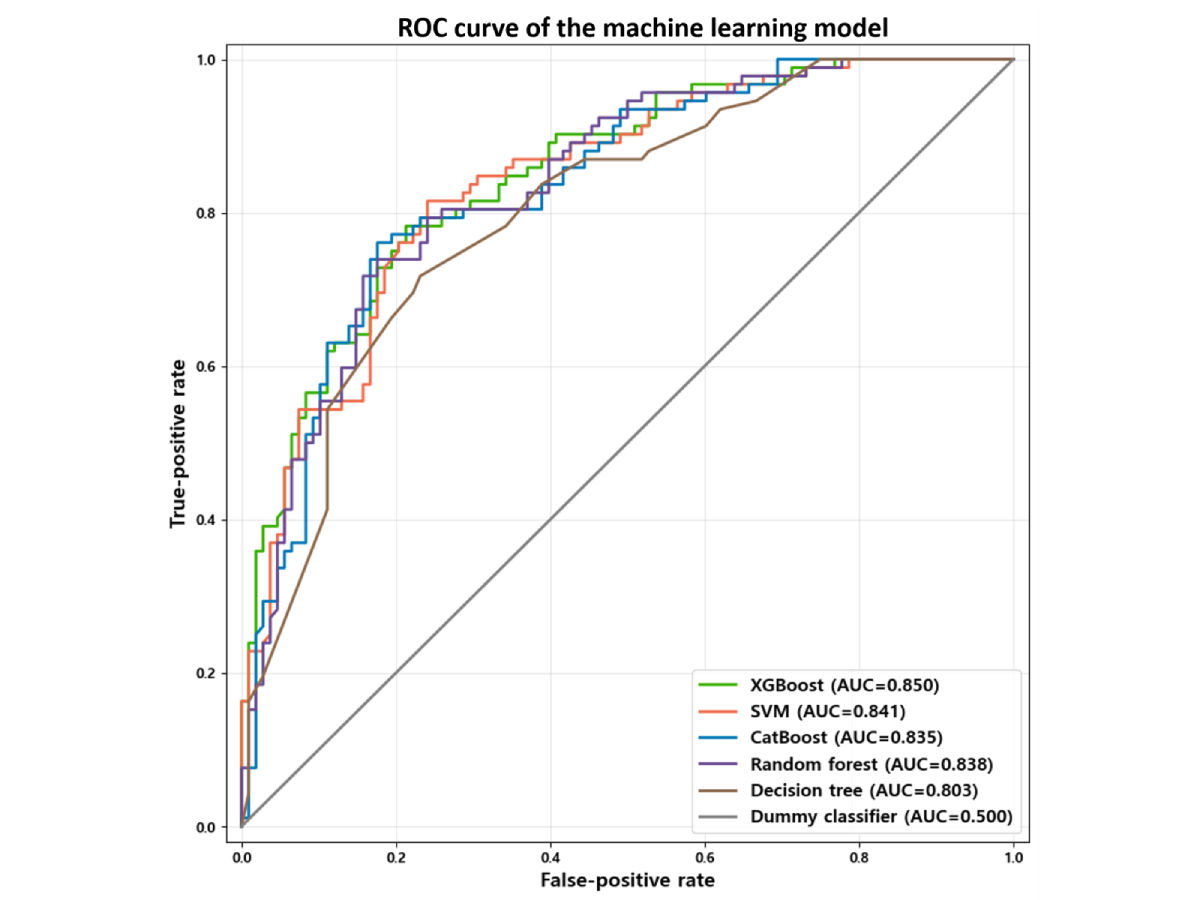
Accuracy and receiver operating characteristic (ROC) curve. AUC: area under the receiver operating characteristic; CatBoost: categorical boosting; SVM: support vector machine; XGBoost: Extreme Gradient Boosting.

The performance of the 5 machine learning models was evaluated and compared using multiple classification metrics, including accuracy, precision, and *F*_1_-score. The detailed results are summarized in Table 4, and the corresponding ROC curves are shown in Figure 3.

To establish a performance baseline given the class distribution, a dummy classifier was evaluated, yielding an accuracy of 0.540. Relative to this baseline, all machine learning models demonstrated improved predictive performance. Among the 5 models, SVM showed strong results, achieving an accuracy of 0.775, a precision of 0.758, and an *F*_1_-score of 0.754. By contrast, the decision tree model exhibited the lowest performance across all metrics, with an accuracy of 0.715, a precision of 0.661, and an *F*_1_-score of 0.716. In addition to standard metrics, ROC curve analysis was conducted to assess the discriminative capability of each model. XGBoost and SVM achieved the highest AUC values (0.850 and 0.841, respectively). Despite these results, CatBoost demonstrated the most balanced and superior overall performance, with an accuracy of 0.785, a precision of 0.769, and an *F*_1_-score of 0.765. Compared with the dummy classifier, CatBoost improved accuracy by 0.245, which was statistically significant (*P*<.001). This consistently strong performance across primary classification metrics constituted the principal rationale for selecting CatBoost for subsequent SHAP-based post hoc interpretability analysis. These findings indicate that, for DHL prediction, the CatBoost model offers robust and reliable classification performance among the algorithms tested.

**Table 4:** Performance metrics of machine learning models.^a^

Model	Accuracy	Precision	Specificity	Sensitivity	*F*_1_-score	Area under the receiver operating characteristic
Dummy classifier	0.540	0.000	1.000	0.000	0.000	0.500
Decision tree	0.715	0.661	0.657	*0.783*	0.716	0.803
Support vector machine	0.775	0.758	0.796	0.750	0.754	0.841
Random forest	0.780	*0.793*	0.843	0.707	0.747	0.838
Extreme gradient boosting	0.780	0.767	0.806	0.750	0.758	*0.850*
Categorical boosting	*0.785*	0.769	*0.806*	0.761	*0.765*	0.835

^a^Values displayed in italics represent the highest performance achieved by each metric when comparing the performance of the 5 machine learning models.

### Model Interpretation Using SHAP Analysis

**Figure 4:**
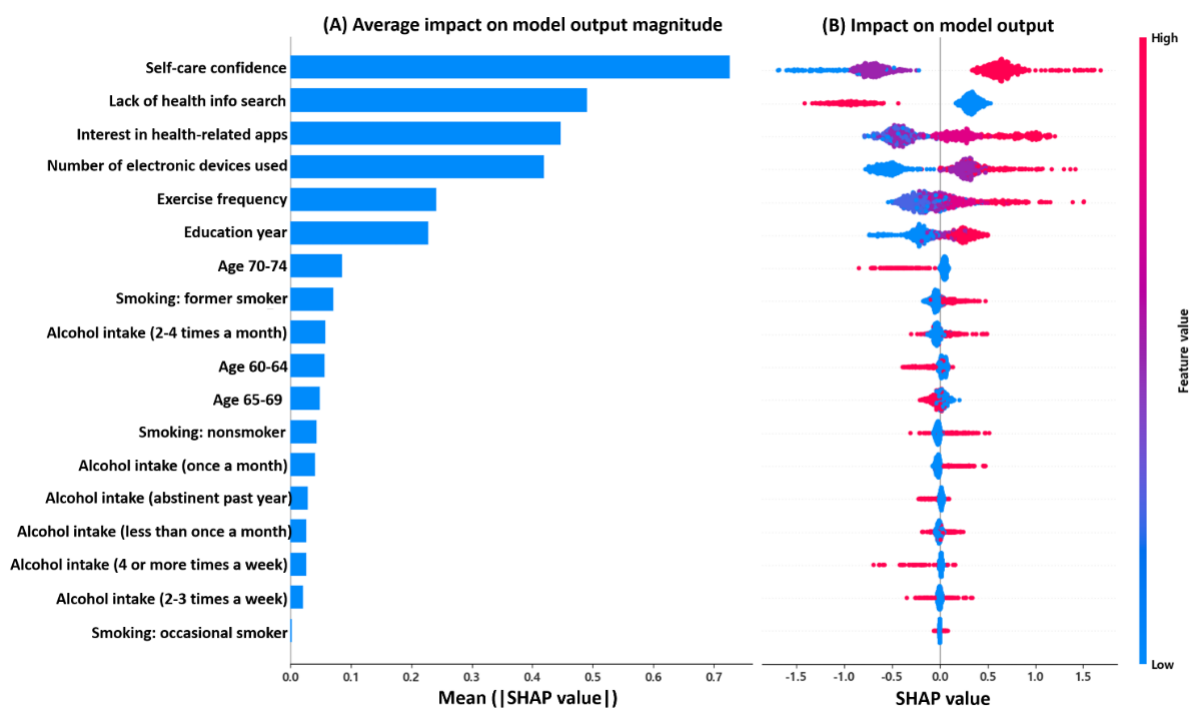
Feature importance based on mean Shapley Additive Explanations (SHAP) values according to the contribution of the categorical boosting model to its estimation. (A) Average impact on model output magnitude; (B) Impact on model output.

The SHAP analysis was conducted to identify the features that contributed most substantially to the model’s predictions of DHL levels. Figure 4A presents the mean absolute SHAP values for each feature, indicating their relative importance within the model. The 5 most influential features were self-care confidence, lack of health information search, interest in health-related apps, number of electronic devices used, and exercise frequency. These variables played a central role in distinguishing between high- and low-DHL classifications.

Figure 4B presents a summary plot illustrating the distribution of SHAP values across different feature values. Self-care confidence emerged as the most influential variable, showing the greatest separation between high- and low-DHL predictions. Higher levels of self-care confidence were associated with positive SHAP values, increasing the likelihood of being classified as high DHL. By contrast, lower self-care confidence levels (represented by blue markers) corresponded to negative SHAP values, contributing to low-DHL predictions.

The binary feature representing lack of health information search, derived from one-hot encoding, also demonstrated a strong effect. Participants who had searched for health information (feature value=0, shown in blue) tended to have positive SHAP values, whereas those who had not (feature value=1, shown in red) exhibited negative SHAP values, indicating a higher likelihood of being classified in the low-DHL group. Similarly, interest in health-related apps and the number of electronic devices used were positively associated with high-DHL predictions. Participants with a greater interest in health-related apps and more frequent use of digital devices showed consistently positive SHAP values, reinforcing their roles as indicators of digital engagement. SHAP values for other contributing factors, including exercise frequency and education level, were relatively small. Age-related features also had comparatively low influence on model predictions; however, the 70-74 age group consistently appeared in association with lower DHL levels, contributing negatively to the prediction outcomes. Overall, the SHAP analysis provided interpretable evidence of how specific behavioral, psychosocial, and demographic features influenced DHL classification, reinforcing both the explainability and practical utility of the CatBoost model in real-world digital health contexts.

## Discussion

### Principal Findings

Digital health literacy, defined as the ability to search for, understand, and apply health-related information in digital environments [4], has emerged as a core competency for effectively navigating modern medical and health care services. As health care delivery models evolve from static information provision to dynamic, user-centered platforms, precise assessment of DHL—particularly among older adults—has become increasingly critical. Accurate measurement of DHL is essential for designing tailored interventions that promote equitable access to health care resources and reduce health disparities.

In this study, we developed an explainable machine learning–based predictive framework to evaluate DHL in a large sample of older adults (n=1000). The framework integrates both performance-based and self-assessed DHL measures and incorporates a range of psychosocial and behavioral variables to identify the most influential predictors contributing to individual differences in digital health literacy.

### Selected Features From Performance-Based Digital Health Literacy

To classify participants into 2 DHL-level groups, explainable features were selected based on the intrinsic evaluation criteria of the Bayesian linear regression model. The analysis revealed that, within the performance-based assessment, key predictors included alcohol intake, number of electronic devices used, and years of education. Among these, the number of electronic devices used emerged as the primary positive predictor of digital information comprehension. This finding is consistent with previous studies showing that older adults who use a wider variety of digital devices are more familiar with online health information and demonstrate stronger skills in information search and utilization [57].

Similarly, years of education positively influenced the comprehension of health information. This finding aligns with previous research identifying educational attainment as a key social determinant of health literacy. Higher educational attainment is generally associated with a greater capacity to comprehend, critically evaluate, and utilize online health information [16,58]. Individuals with higher levels of education are relatively more advantaged in discerning the credibility of health information and translating it into effective communication with health care providers or self-management behaviors, which in turn facilitates greater acceptance and utilization of digital health services [59,60]. A comparative study involving older adults in Korea and the United States likewise identified educational attainment as a consistent predictor of eHealth literacy, while noting that its impact may be moderated by structural and cultural factors within the digital environment [15]. Collectively, these findings underscore the importance of designing digital health interventions that account for educational disparities and incorporate tailored capacity-building strategies for individuals with lower levels of formal education.

By contrast, participants with higher levels of alcohol consumption exhibited significantly lower performance in both digital and health information comprehension, suggesting that lifestyle factors such as alcohol intake may impair information-processing abilities and negatively affect DHL. Moreover, a previous longitudinal study reported that sustained alcohol consumption is associated with cognitive decline, particularly among older women [61]. Taken together, these results indicate that diversity in digital device usage and higher educational attainment contribute meaningfully to actual information comprehension, and that lifestyle behavior management should be considered a critical component of DHL intervention programs.

### Selected Features From Self-Assessed Digital Health Literacy

Self-assessed digital health literacy was primarily influenced by interest in health-related apps, age, alcohol intake, number of electronic devices used, and years of education. Additional contributing factors included self-care confidence, smoking status, and exercise frequency. Specifically, perceived competence in using digital devices was associated with greater confidence in health care decision-making, whereas the ability to understand and apply health information was linked to participants’ prior health information–seeking behavior.

Among these variables, interest in health-related apps emerged as the most prominent positive predictor of both DHL subscales. Furthermore, a previous study reported that individuals who frequently engage with health care apps and perceive them as useful or reliable tend to demonstrate stronger competencies in locating, evaluating, and applying health information [57]. Interest in health-related apps could reflect more than mere preference, as it represents a motivational factor that enhances digital engagement. Voluntary use of such apps may foster critical components of DHL, including information searching, selection, appraisal, and application. Therefore, interventions designed to improve DHL should extend beyond digital tools and unidirectional health messaging.

In this study, we found that the older the age group, the lower their tendency to evaluate their understanding of and ability to use health information. It has been reported that older adults display a reduced ability to adopt digital technology because of cognitive decline [62]. Moreover, previous studies have found that older adults living alone face both cognitive and emotional barriers when adapting to new technologies [63-67]. Addressing these barriers requires tailored educational strategies that combine practical digital training with emotional support.

The number of electronic devices used and years of education proved to be negatively associated with self-assessed DHL. This finding suggests that greater exposure to digital devices or higher educational attainment does not necessarily translate into higher self-assessed DHL. The strength and nature of the relationship between education and digital health literacy may vary depending on the sociocultural context and the health care and digital infrastructure [68]. In addition, individuals with greater actual abilities may apply stricter self-assessment criteria, reflecting more conservative or critical self-assessment tendencies. A prior finding indicated that older adults may experience increased cognitive load when processing complex health-related information, leading to reduced confidence and lower self-ratings [69].

A similar pattern was observed for health information search. In the feature selection stage, lack of health information search was positively associated with the health information understanding and utilization subscale in the self-assessed survey. By contrast, in the model interpretation stage, the same indicator showed negative SHAP values, thereby decreasing the model’s predicted likelihood of high DHL. Individuals who do not search for health information may positively assess their understanding of health information even though they lack it, whereas health information seekers might provide cautious self-assessments despite possessing substantial digital health literacy skills. It is known that self-reported responses do not represent a direct readout of an individual’s internal state but are rather the product of complex cognitive processes such as comprehension, retrieval, judgment, and response selection, potentially leading to biased or distorted outcomes [70]. These findings emphasize the constraints of self-reported measures and the imperative to validate subjective data with objective performance metrics [71].

In addition, alcohol intake showed a clear divergence by measurement modality. In the self-assessed digital device utilization subscale, alcohol-related categories were positively associated with utilization scores, including abstinence in the past year, alcohol intake about once a month, and alcohol intake 2-4 times a month. By contrast, in the performance-based DHL tasks, alcohol intake once a month and alcohol intake 2-4 times a month were associated with lower task performance. Prior research in older adults indicates that internet use does not consistently correspond to healthier behaviors and that associations may vary by socioeconomic and lifestyle profiles [72]. A population-based study using CHARLS (The China Health and Retirement Longitudinal Study) 2020 data reported that internet use was associated with a lower likelihood of nondrinking, and this association was partially mediated by social participation [73]. This suggests that digital access may increase opportunities for social activities in which alcohol intake is culturally embedded. Taken together, performance-based DHL tasks are more closely tied to the cognitive and informational demands of digital health activities and may be more sensitive to alcohol-related decrements. By contrast, self-assessed digital device utilization may reflect a broader lifestyle context rather than objective capability alone.

### Development of the Prediction Models

The first stage of the DHL framework identified variables with high explanatory power for both performance-based and self-assessed measures. In the second stage, these selected variables were used to train models predicting DHL levels based on KeHEALS. The model performance evaluation demonstrated that the CatBoost algorithm consistently outperformed the alternative approaches, achieving comparatively high accuracy, precision, and *F*_1_-scores in predicting DHL among older adults (Table 3). Compared with a conventional single decision tree, CatBoost, which effectively handles categorical variables, proved particularly effective for DHL prediction.

To enhance interpretability, a SHAP analysis was conducted on the CatBoost model, providing empirical evidence of the specific factors influencing DHL (Figure 4). The most influential predictors included self-care confidence, lack of health information search, interest in health-related apps, number of electronic devices used, exercise frequency, and years of education. This confirms that the framework successfully integrates both digital and health literacy–related features into a unified predictive model. Among the key predictors, self-care confidence emerged as the single most important predictor, highlighting the strong association between an individual’s confidence in making health-related decisions and their DHL level. This finding aligns with prior research reporting that higher eHealth literacy is significantly associated with greater self-efficacy in older adults [74]. This evidence underscores the potential of DHL-focused interventions to enhance broader health management competencies by strengthening decision-making confidence. Furthermore, consistent with the results of the self-assessed DHL, interest in health-related apps reflected an individual’s proactive attitude toward information seeking and app utilization, reinforcing the importance of psychosocial factors alongside technical competencies. Accordingly, interventions aimed at improving DHL should combine technical skill development with psychosocial support to enhance motivation for and engagement with digital health tools [75].

In contrast to the preceding factors, a greater number of electronic devices used, higher exercise frequency, and higher educational attainment were paradoxically associated with lower self-assessed scores for use of digital devices and health information understanding and utilization. These results may reflect the subjective biases inherent in self-reports. Self-report measures are constrained by their susceptibility to respondents’ memory and cognitive processes, social desirability and consistency motives, and instrument design features such as item wording, response scales, and question order [76-78]. Alcohol intake showed a similar discrepancy. In the self-assessed digital device utilization subscale, abstinence in the past year and infrequent drinking were positively associated with utilization scores. By contrast, similar frequencies of alcohol intake were associated with lower performance in the task-based DHL assessment. This pattern suggests that self-assessed utilization may reflect a broader socioeconomic and lifestyle context, whereas performance-based tasks reflect the cognitive and informational demands required for digital health activities.

Therefore, a multimethod approach that integrates both self-assessments and performance-based evaluations is essential for a comprehensive understanding of DHL, rather than relying solely on self-report data. When variables derived from both measures were incorporated into the machine learning model, SHAP analysis indicated that these factors positively contributed to the classification of the high-DHL group, corroborating the results of the performance-based assessment. This demonstrates that reliance on self-reported data alone may lead to biased inferences or the overlooking of important patterns. By combining multifaceted indicators with explainable machine learning techniques, our study’s approach enables more precise identification of the determinants of DHL in older adults and provides practical evidence to inform the design of individualized digital health interventions.

KeHEALS is a broadly used and validated assessment tool, although it yields only a single metric for digital literacy. Therefore, it remains uncertain whether individual vulnerabilities stem from fundamental digital device use, comprehension and appraisal of health information, or demographic and psychosocial factors. By using the detailed subscales evaluated within the DHLS, we can elucidate why a particular individual falls into the low-DHL category according to multidimensional criteria. When these domain-informed features are used to predict KeHEALS levels and the resulting predictions are subsequently examined using SHAP, KeHEALS scores can be analytically decomposed into discrete contributions originating from specific digital and health domains, along with additional contextual factors. This study highlights the limitations of single-score screening instruments and advances personalized interventions by offering explanations of feature importance through SHAP analysis.

This study leverages both performance- and survey-based datasets to bridge the gap between self-reported and actual abilities. Using Bayesian regression, the variables explaining each dimension were identified and integrated into the final DHL prediction model. SHAP analysis further validated the consistency between variable importance in the feature-selection stage and the variables’ impact on model learning. This dual-phase approach enabled both ante hoc interpretability—by incorporating transparency into the model design from the outset—and post hoc interpretability, addressing the “black box” issue while retaining explanatory power. Finally, the DHL framework offers practical guidance for assessing users’ DHL before implementing digital health interventions. This can be particularly valuable in clinical contexts where evaluation and interpretability are critical, thereby supporting the design of tailored and effective health care strategies for older adults.

### Limitations

This study has several limitations that should be noted. First, the performance-based features were identified from a pilot cohort aged 60-74, whereas the predictive model was trained on a wider age range (55-74 years). Our post hoc comparison revealed that several selected variables differed significantly between the 55-59 and 60-74 age groups, suggesting meaningful demographic and behavioral heterogeneity across age groups. Therefore, the generalizability of the feature-selection process from the small pilot cohort to the younger subgroup may be limited. Future research should employ larger and more diverse samples to enhance the robustness and external validity of the performance-based assessments. Second, because all data collected focused exclusively on older adults in Korea, caution should be exercised when extrapolating the results to populations from different cultural and demographic contexts. Third, the behavioral task was conducted using a single Android smartphone, and all participants were Android users. Thus, familiarity with the Android interface may have been reflected in the digital information comprehension scores and may not fully capture device proficiency among iOS users or across heterogeneous device environments. Fourth, there are inherent limitations related to the measurement scales and variable-processing strategies employed in this study. While offering interpretive simplicity, the binary classification of DHL levels may lead to different outcomes if alternative KeHEALS score thresholds are applied. Furthermore, self-care confidence, which emerged as the primary driver in our predictive model, was assessed using a single-item measure derived from the Korean Health Empowerment Scale. As single-item measures typically exhibit lower reliability and less comprehensive construct coverage than multiitem scales, the high predictive importance of this variable should be interpreted with caution. Additionally, our feature-importance rankings may have been influenced by the mixed encoding strategies used during data preprocessing. Specifically, the use of one-hot encoding for certain lifestyle factors, such as alcohol intake, partitioned these variables into multiple binary features. This approach may have diluted their aggregate predictive power in the SHAP analysis, potentially leading to an artificial deflation of their importance compared with variables maintained as single ordinal features. Finally, actual DHL is likely to exist along a continuous and multifaceted spectrum. Features that demonstrated unexpected associations in the self-assessed DHL analysis, such as the number of electronic devices used, exercise frequency, and years of education, may reflect the inherent limitations of self-report measures or complex interaction effects with other variables. These observations underscore the need for further in-depth analyses to disentangle these relationships.

### Conclusions

DHL is a key competency for older adults to effectively navigate modern health care. This study established an explainable machine learning framework that integrates performance-based and self-assessed measures to evaluate DHL. Our findings indicate that using a wider variety of electronic devices and having higher educational attainment were positively associated with performance-based DHL, whereas higher alcohol intake was linked to poorer comprehension. Given the limited sample size of the performance-based pilot, these associations warrant caution and require validation in larger cohorts. In the self-assessed domain, DHL levels were primarily related to interest in health-related apps, age, and self-care confidence. In the predictive modeling stage, the CatBoost model demonstrated superior performance, and SHAP analysis identified self-care confidence and interest in health-related apps as key contributors to high DHL. Overall, by combining performance-based and self-assessed measures, this framework offers a structured and interpretable approach to assessing DHL in older adults and can inform the design of tailored digital health interventions in clinical and community settings.

## Appendix

Multimedia Appendix 1 Distribution of digital literacy and health literacy scores by values of features.

## Abbreviations

AUC: area under the receiver operating characteristic
CatBoost: categorical boosting
CHARLS: The China Health and Retirement Longitudinal Study
DHL: digital health literacy
DHLS: Digital Health Literacy Scale
eHEALS: eHealth Literacy Scale
HDI: highest density interval
KeHEALS: Korean version of the eHealth Literacy Scale
mHealth: mobile health
ROC: receiver operating characteristic
SHAP: Shapley Additive Explanations
SVM: support vector machine
XGBoost: Extreme Gradient Boosting

## Data Availability

The data and code underlying this article will be shared on reasonable request to the corresponding author (JWC).
