# Supplementary material for: Explainable Machine Learning for Assessing Digital Health Literacy in Older Adults: Validation and Development of a Two-Stage Model Integrating Performance-Based and Self-Assessed Indicators
Source: JMIR Med Inform. 2026 Mar 23;14:e86171. doi: 10.2196/86171 (PMC13054219; doi:10.2196/86171)

Multimedia Appendix 1

Figure 1. Distribution of digital literacy scores by values of features


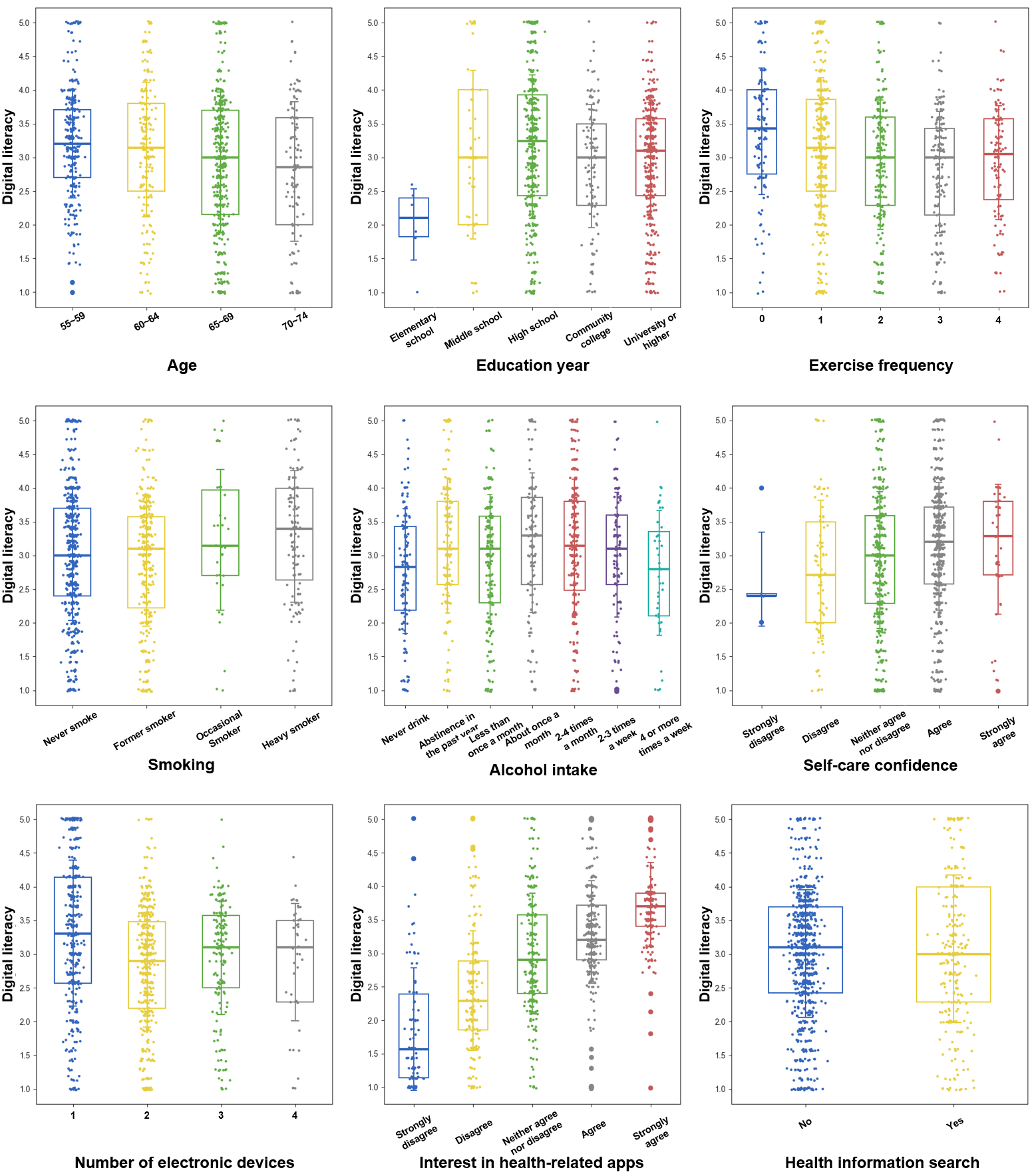


Figure 2. Distribution of health literacy scores by values of features


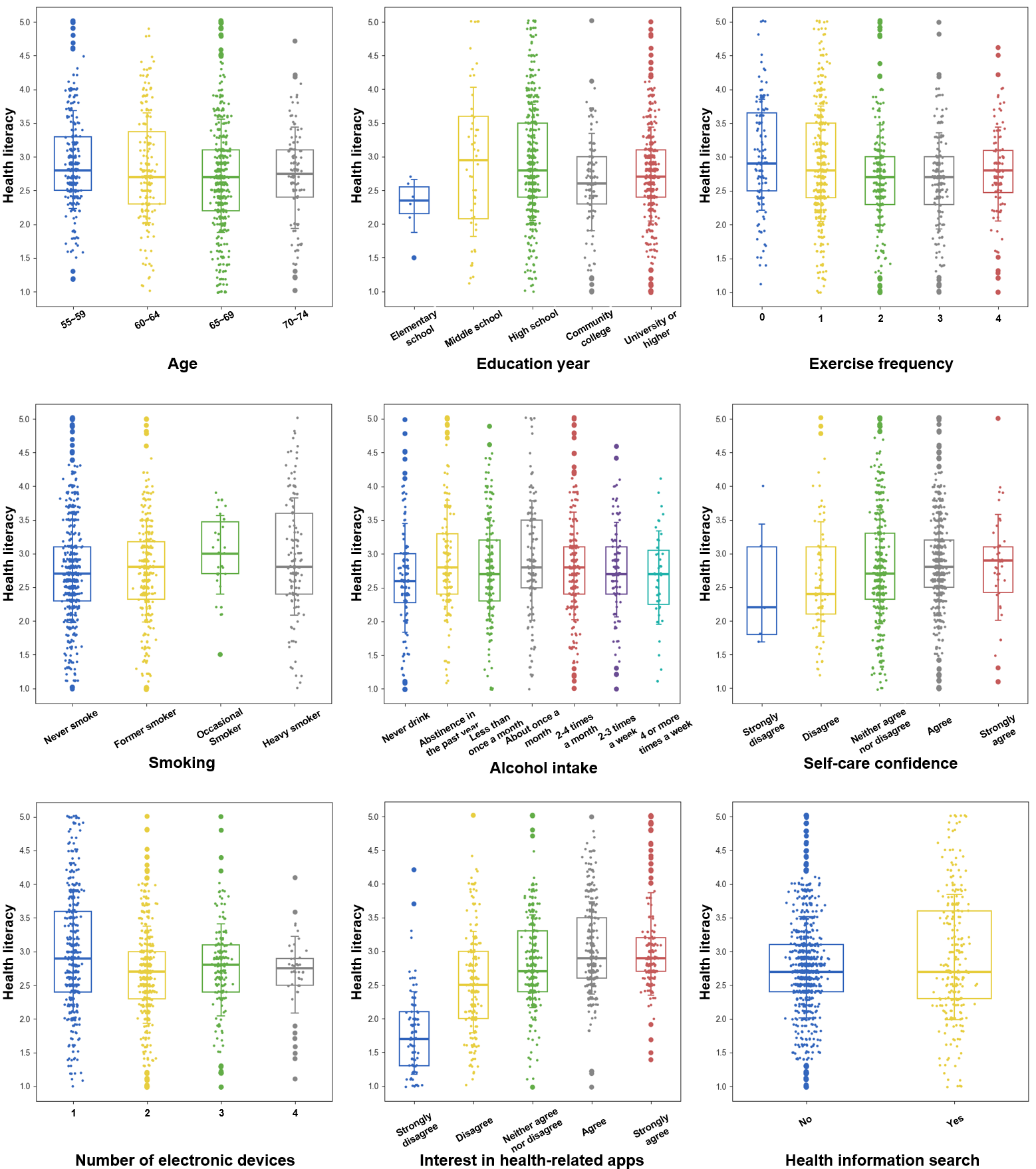

Supplement: Multimedia Appendix 1 [file medinform_v14i1e86171_app1.docx]
